# Decreased striatal dopamine in group II metabotropic glutamate receptor (mGlu2/mGlu3) double knockout mice

**DOI:** 10.1186/1471-2202-14-102

**Published:** 2013-09-22

**Authors:** Tracy A Lane, Thomas Boerner, David M Bannerman, James NC Kew, Elizabeth M Tunbridge, Trevor Sharp, Paul J Harrison

**Affiliations:** 1 Department of Psychiatry, University of Oxford, Warneford Hospital, Oxford OX3 7JX, UK; 2 Department of Experimental Psychology, University of Oxford, Oxford, UK; 3 Neuroscience CEDD, GlaxoSmithKline, Harlow, UK; 4 Department of Pharmacology, University of Oxford, Oxford, UK

**Keywords:** mGlu2, mGlu3, Grm2, Grm3, HPLC, Striatum, Nucleus accumbens

## Abstract

**Background:**

Group II metabotropic glutamate receptors (mGlu2 and mGlu3, encoded by *Grm2* and *Grm3*) have been the focus of attention as treatment targets for a number of psychiatric conditions. Double knockout mice lacking mGlu2 and mGlu3 (mGlu2/3^−/−^) show a subtle behavioural phenotype, being hypoactive under basal conditions and in response to amphetamine, and with a spatial memory deficit that depends on the arousal properties of the task. The neurochemical correlates of this profile are unknown. Here, we measured tissue levels of dopamine, 5-HT, noradrenaline and their metabolites in the striatum and frontal cortex of mGlu2/3^−/−^ double knockout mice, using high performance liquid chromatography. We also measured the same parameters in mGlu2^−/−^ and mGlu3^−/−^ single knockout mice.

**Results:**

mGlu2/3^−/−^mice had reduced dopamine levels in the striatum but not in frontal cortex, compared to wild-types. In a separate cohort we replicated this deficit and, using tissue punches, found it was more prominent in the nucleus accumbens than in dorsolateral striatum. Noradrenaline, 5-HT and their metabolites were not altered in the striatum of mGlu2/3^−/−^ mice, although the noradrenaline metabolite MHPG was increased in the cortex. In mGlu2^−/−^ and mGlu3^−/−^ single knockout mice we found no difference in any monoamine or metabolite, in either brain region, compared to their wild-type littermates.

**Conclusions:**

Group II metabotropic glutamate receptors impact upon striatal dopamine. The effect may contribute to the behavioural phenotype of mGlu2/3^−/−^ mice. The lack of dopaminergic alterations in mGlu2^−/−^ and mGlu3^−/−^ single knockout mice reveals a degree of redundancy between the two receptors. The findings support the possibility that interactions between mGlu2/3 and dopamine may be relevant to the pathophysiology and therapy of schizophrenia and other disorders.

## Background

Group II metabotropic glutamate receptors, mGlu2 and mGlu3, are G protein coupled receptors that negatively regulate adenylate cyclase [1]. The receptors are predominantly located pre-synaptically, where they function as auto- and hetero-receptors, inhibiting release of glutamate and other neurotransmitters, including dopamine. mGlu2 and mGlu3 are widely expressed in the brain with overlapping but not identical regional, cellular, and subcellular distributions [2].

mGlu2 and mGlu3 have been implicated in a number of psychiatric disorders including, schizophrenia, addiction, anxiety and depression. In schizophrenia, for example, allelic variation in *GRM3* (the gene encoding mGlu3) has been associated with disease risk, and mGlu2/3 agonists have shown efficacy in models of the disorder, and in one study, of the disorder itself [3-5]. However, the overall picture is complex, with studies showing varying results, and with uncertainty as to the pathways by which mGlu2/3 operate, including how they impact upon dopamine, the neurotransmitter central to existing treatments for schizophrenia.

Knockout mice lacking mGlu2 (mGlu2^−/−^) or mGlu3 (mGlu3^−/−^) have been generated to help characterize the function of these receptors. Results emphasize that the roles of these receptors are subtle, with the mice showing no overt behavioural or neurochemical phenotype at baseline [6-8]. This may in part be due to compensatory up-regulation of the remaining receptor [9]. Double knockout mice lacking both mGlu2 and mGlu3 (mGlu2/3^−/−^; also known as Grm2/3^−/−^) show an emergent, and complex, phenotype. Specifically, the mice are hypoactive, are less sensitive to amphetamine induced hyperactivity, and have a spatial memory impairment in appetitive but not aversive tasks [10].

The neurochemical correlates of these behavioural findings are unknown, although the hypoactivity at baseline and in response to amphetamine are suggestive of alterations in dopamine and potentially of other monoamines. This is of relevance to the roles which group II mGlus may play in, or as targets for, dopamine-related psychiatric disorders. Here, we used high performance liquid chromatography (HPLC) to measure tissue levels of monoamines and their metabolites in the cortex and striatum of mGlu2/3^−/−^ mice. Given the findings, we repeated the study in mGlu2^−/−^ and mGlu3^−/−^ single knockout mice, and also measured dopamine separately in the ventral striatum (nucleus accumbens [NAc]) and dorsolateral striatum of the mGlu2/3^−/−^ mice.

## Results

**Figure 1:**
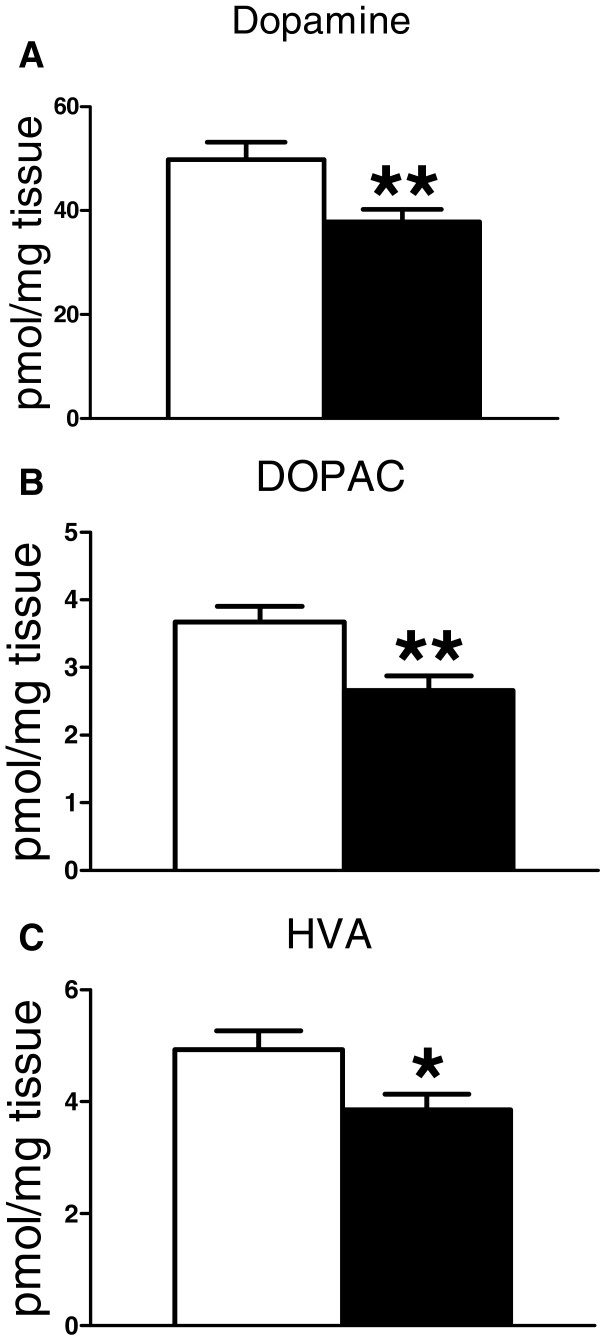
**Dopamine and dopamine metabolites are reduced in the striatum of mGlu2/3**^**-/- **^**mice. (A)** Dopamine content is reduced in the striatum of mGlu2/3^-/-^ mice (black) compared to wildtype mice (white), n = 14 WT, 16 KO. Dopamine metabolites DOPAC **(B)** and HVA **(C)** are also reduced. Monoamine concentration is normalised to the tissue weight in mg. Graphs show mean ± SEM; all one-way ANOVA, *p=0.023; **p<0.005.

Tissue monoamine levels were measured in mGlu2/3^−/−^ and wild-type mice. The main finding is a reduction in dopamine content in the striatum (~25%, p=0.005), accompanied by similar reductions in the metabolites DOPAC (~28%, p=0.004) and HVA (~22%, p=0.023; Figure 1) of the mGlu2/3^−/−^ mice. In addition, the noradrenaline metabolite MHPG was increased (~31%, p=0.003) in frontal cortex (Table 1). There were no significant differences between groups in cortical dopamine metabolism, nor in 5-HT or its metabolite 5-HIAA in either region (Table 1). No significant interactions of sex and genotype were found.

**Table 1:** **Monoamines and their metabolites in the cortex and striatum of mGlu2/3**^**−/− **^**double knockout and wild-type (WT) mice**

	**Cortex**		**Striatum**	
	**mGlu2/3 WT**	**mGlu2/3**^**−/−**^	**mGlu2/3 WT**	**mGlu2/3**^**−/−**^
Dopamine	1.7 +/− 0.51	1.4 +/− 0.34	49.8 +/−3.38	37.8 +/− 2.40^a^
DOPAC	0.6 +/− 0.13	0.5 +/− 0.09	3.8 +/− 0.23	2.7 +/− 0.22^b^
HVA	0.8 +/− 0.10	0.9 +/− 0.12	4.9 +/− 0.33	3.9 +/− 0.28^c^
Noradrenaline	1.6 +/− 0.08	1.8 +/− 0.07	1.4 +/− 0.07	1.6 +/− 0.15
MHPG	2.2 +/− 0.17	2.9 +/− 0.14^d^	4.3 +/− 0.38	4.2 +/− 0.41
5HT	2.9 +/− 0.13	3.1 +/− 0.09	3.5 +/− 0.20	3.6 +/− 0.23
5HIAA	1.1 +/− 0.08	1.3 +/− 0.08	1.8 +/− 0.11	1.8 +/− 0.12

n=12-16 mGlu2/3^−/−^ mice, and 12–14 WT mice. Values are pmol/mg tissue, mean ± SEM.

^a^F1,26 = 9.25, p=0.005.

^b^F1,26 = 9.85, p=0.004.

^c^F1,26 = 5.84, p=0.023.

^d^F1,24 = 11.1, p=0.003.

The experiment was repeated in single mGlu2^−/−^ and mGlu3^−/−^ knockout mice and their respective littermate controls. As detailed in Table 2, there were no differences in monoamines or their metabolites between genotypes. In particular, striatal dopamine showed no differences, nor trends.

**Table 2:** **Monoamines and their metabolites in the cortex and striatum of mGlu2**^**−/− **^**and mGlu3**^**−/− **^**mice and their respective wild-type (WT) littermates**

	**mGlu2 WT**	**mGlu2**^**−/−**^	**mGlu3 WT**	**mGlu3**^**−/−**^
	Cortex			
Dopamine	0.5 +/− 0.08	0.4 +/− 0.07	0.4 +/− 0.04	0.4 +/− 0.05
DOPAC	0.2 +/− 0.03	0.2 +/− 0.01	0.2 +/− 0.02	0.2 +/− 0.03
Noradrenaline	2.0 +/− 0.18	1.8 +/− 0.16	2.2 +/− 0.19	2.3 +/− 0.22
MHPG	0.6 +/− 0.05	0.6 +/− 0.05	0.7 +/− 0.10	0.8 +/− 0.07
5-HT	3.1 +/− 0.19	3.1 +/− 0.23	3.1 +/− 0.20	2.9 +/− 0.25
5-HIAA	1.0 +/− 0.03	0.8 +/− 0.06	0.8 +/− 0.08	0.7 +/− 0.06
	Striatum			
Dopamine	34.0 +/− 3.8	36.5 +/− 3.8	35.7 +/− 4.7	32.6 +/− 3.3
DOPAC	2.3 +/− 0.2	2.4 +/− 0.2	2.1 +/− 0.1	2.2 +/− 0.2
HVA	2.8 +/− 0.2	3.1 +/− 0.2	2.3 +/− 0.2	3.0 +/− 0.3
Noradrenaline	2.0 +/− 0.2	1.8 +/− 0.2	1.5 +/− 0.1	1.6 +/− 0.1
5-HT	2.9 +/− 0.2	3.1 +/− 0.2	3.6 +/− 0.2	3.5 +/− 0.3
5-HIAA	1.6 +/− 0.1	1.6 +/− 0.1	1.6 +/− 0.1	1.8 +/− 0.03

n=6-8 in each group. Values are pmol/mg tissue, mean ± SEM. There are no significant differences between groups. Reliable measurements for cortical HVA and striatal MHPG could not be obtained.

**Figure 2:**
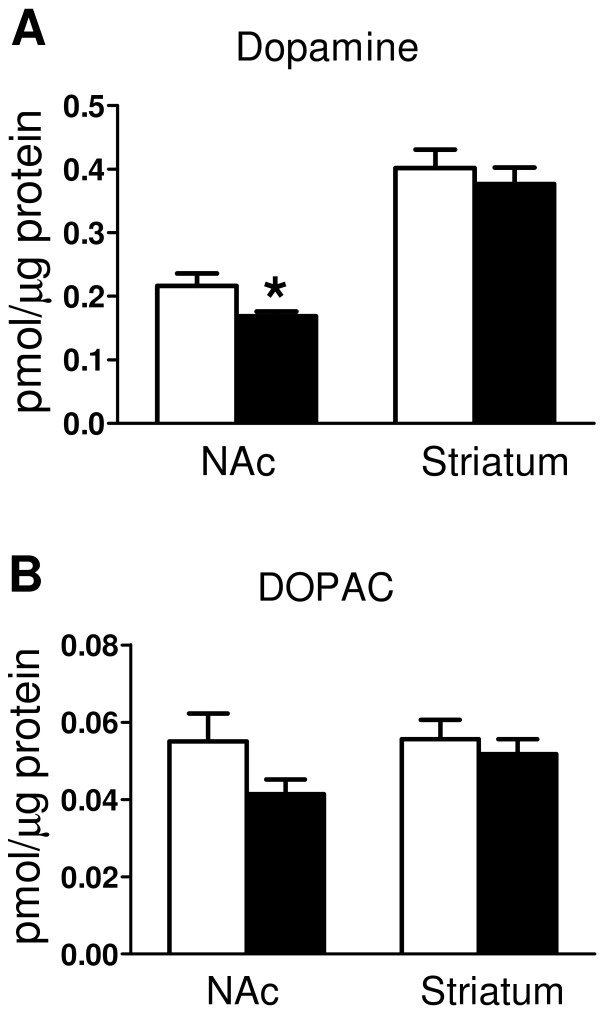
**Dopamine is reduced in tissue punches of the NAc but not dorsolateral striatum of mGlu2/3**^**-/- **^**mice. A**: Dopamine content is reduced in tissue punches of NAc but not dorsolateral striatum of mGlu2/3^-/-^ mice (black) compared to wildtypes (white). NAc n = 12 WT, 12 KO and dorsolateral striatum n=12 WT, 14 KO. **B**: DOPAC shows a trend towards reduction in the NAc of mGlu2/3-/- mice (p=0.1). NAc n = 12 WT, 14 KO and dorsolateral striatum n= 12 WT, 15 KO. Monoamine concentration is normalised to the total protein content of the punches. Graphs show mean ±SEM; all one-way ANOVA except striatal DOPAC, analysed with Mann Whitney U test, *p=0.037.

To replicate and better characterize the striatal dopamine findings seen in the mGlu2/3^−/−^ mice, a second cohort of animals were studied, from which tissue micropunches were taken to allow separate measurements in dorsolateral striatum and NAc. Figure 2 shows that dopamine content was decreased in NAc (~22%, p=0.037) but not in the dorsolateral striatum (~6%, p=0.837) of mGlu2/3^−/−^ mice. DOPAC followed a similar trend (NAc ~25% p=0.1, and striatum ~7%, p=0.98); HVA could not be measured in this experiment.

## Discussion

Interactions between mGlu2/3 and monoamines, particularly dopamine, are of interest because of the putative roles of these receptors in the pathophysiology and therapy of various dopamine-related psychiatric disorders, including schizophrenia, anxiety, and addiction. The main finding of the present study is that deletion of both receptors leads to a decrease in striatal dopamine, particularly in the NAc. The data complement the many previous pharmacological studies which have shown effects of mGlu2/3 agonists and antagonists on dopamine, albeit with complex and in places inconsistent results [4,11-21].

mGlu2/3^−/−^ mice are hypoactive and show decreased responsiveness to amphetamine [10]. It is plausible that the reduced dopamine ‘tone’ reported here contributes to these findings. Whether it also underlies the arousal-dependent effect on spatial memory [10] or the impaired long-term depression [22] of the mice, is unknown. But it may be significant that the NAc is involved in motivation, reward and aversion [23,24]. Noradrenaline also plays a role in attention, arousal and stress [25] and mGlu2/3 ligands have been shown to regulate NA release [26]. We observed an increase in cortical MHPG and an increase in the MHPG to noradrenaline ratio (p=0.053), which may reflect an increase in noradrenaline turnover and reduced pre-synaptic α2-adrenoceptor activity [27], therefore it is possible that changes in noradrenaline signalling are also important for the behavioural findings in these mice. In a future study, simultaneous behavioural and neurochemical measurements could be taken, helping to confirm and clarify the links between them, and could be extended to include other transmitters, such as GABA and glutamate.

We found no differences in monoamine content in either mGlu2^−/−^ or mGlu3^−/−^mice. In this respect the findings in the mGlu2/3^−/−^ mice were emergent, and mirror the behavioural profile, which is present in the mGlu2/3^−/−^ mice [10] but is not observed in either single knockout line [6-8,28-30] (and unpublished observations; De Filippis, DMB, PJH, TAL). One relevant finding in the mGlu2^−/−^ mice is that despite no changes in striatal tissue levels of dopamine (current data and [7]) and no baseline dialysate changes in glutamate or dopamine [7], these mice exhibit enhancement of cocaine-induced extracellular dopamine and glutamate in the NAc [7]. There is also evidence that a greater proportion of dopamine D2 receptors are in a high-affinity state in mGlu2^−/−^ and mGlu3^−/−^mice, which may be indicative of a subtle difference in dopaminergic function [31]. By the same token, the unchanged baseline levels of noradrenaline, 5-HT and cortical dopamine in mGlu2/3^−/−^ mice reported here do not preclude the occurrence of functionally significant alterations in monoamine signalling.

The striatal dopamine deficit observed in the mGlu2/3^−/−^ mice could be a result of reduced synthesis and storage, and/or decreased release. The latter explanation seems more likely given that dopamine and its metabolites were all comparably reduced. However, measurements of extracellular dopamine using microdialysis or voltammetry would be needed to confirm this interpretation.

There are a number of locations at which mGlu2/3 may regulate striatal dopamine, either directly or indirectly. One possibility is that the receptors are expressed by, and function within, dopamine neurons. However, neither mGlu2 nor mGlu3 mRNA is detected in the substantia nigra pars compacta, while in the ventral tegmental area (VTA), only very low levels of mGlu2 and no mGlu3 mRNA is detected [32-34]. These data are complemented by findings that mGlu2/3 immunoreactivity does not co-localize with tyrosine hydroxylase positive axons in the striatum [35]. Similarly, there is little or no mGlu2/3 immunoreactivity detected in the VTA or substantia nigra pars compacta [35-37] suggesting that mGlu2/3 are unlikely to be located presynaptic to the dendrites or soma of dopamine neurons. This possibility cannot be excluded entirely since some data have shown functional mGlu2/3 receptors in VTA [38-40]. Nevertheless, together these results suggest that mGlu2/3 regulation of dopamine is not mediated by receptors expressed or located within the dopaminergic nuclei.

Instead, a more likely site for mGlu2/3-mediated modulation of striatal dopamine is within the striatum itself. The striatal neuropil shows strong mGlu2/3 immunoreactivity [2,35-37], accompanied by low to moderate levels of mGlu2 and mGlu3 mRNAs [32-34]. This combination of mRNA and protein data suggests that regulation of striatal dopamine by mGlu2/3 occurs via receptors which are expressed on the axonal processes of neurons projecting in from outside the striatum. Corticostriatal afferents are a strong candidate: mGlu2/3 immunoreactivity is present on their terminals [37,41], striatal mGlu2/3 immunoreactivity is markedly decreased by decortication [35], and there is abundant mGlu2 and mGlu3 mRNA in cortical neurons [32,33,41]. Striatal mGlu2/3 are located at axo-dendritic synapses [42], ruling out direct effects on dopaminergic axon terminals. Thus, mGlu2/3 regulation of striatal dopamine is likely to be mediated via an intrinsic striatal neuron population that does not itself express mGlu2/3. This issue can be clarified by studies of the circuitry underlying the neurochemical results reported here. As part of this work, the possible contribution of glial mGlu3 should be considered [2,35,43], and it will also be necessary to investigate the basis for the preferential reduction in dopamine in NAc versus dorsolateral striatum. Regarding the latter point, it might have been expected that the reduction in dopamine in the NAc (Figure 2) would have been of greater magnitude than that seen in the striatum overall (Figure 1). In the event, both were comparable (22-25%). This may reflect the different methodologies and normalization methods used in the tissue punch and homogenisation experiments, or simply variation between cohorts.

As noted earlier, there have been many pharmacological studies showing that mGlu2/3 agonists and antagonists affect dopamine turnover, release and function [4,11-21]. In this respect our data are consistent with these findings. However, direct comparisons with the earlier pharmacological studies are difficult, for two reasons. Firstly, the most pertinent drug studies would be those using chronic administration of mGlu2/3 antagonists, yet to our knowledge these have not been conducted – the majority of studies have used agonists, and have used acute administration. Secondly, the pharmacological findings have been inconsistent, in terms of the magnitude and even the direction of effects on dopamine levels or release caused by acute administration of mGlu2/3 ligands [4,11-21]. Despite these issues, the present data support, and extend, the evidence that mGlu2/3 impact on dopamine, and thereby may be valuable therapeutic targets for disorders involving dopamine dysregulation.

## Conclusions

Baseline tissue levels of dopamine and dopamine metabolites are reduced in the striatum of mGlu2/3^−/−^ mice, providing novel evidence that group II metabotropic glutamate receptors influence the dopamine system. The finding is relevant to the development of mGlu2/3 ligands as potential therapies for neuropsychiatric disorders. No changes were observed in the frontal cortex, nor in mice lacking only one of the two receptors. The mechanisms by which mGlu2/3^−/−^ deletion results in decreased striatal dopamine (and whether it is a direct or indirect effect) are unknown. They may be clarified by dynamic measures of dopamine functioning, and by investigation of the molecular correlates of the neurochemical changes reported here.

## Methods

All animal studies were carried out in accordance with Animals (Scientific Procedures) Act 1986 and the GSK Policy on the Care, Welfare and Treatment of Animals, and were approved by the relevant local ethics committee.

### Knockout mice

mGlu2 (*Grm2*), mGlu3 (*Grm3*) single knockout (mGlu2^−/−^ or mGlu3^−/−^) and double knockout mice (mGlu2/3^−/−^) on a C57BL/6J background were obtained from GlaxoSmithKline, Harlow, U.K. The mGlu2^−/−^ mice were generated as in [30], mGlu3^−/−^ mice were generated as in [44] and mGlu2/3^−/−^ were generated as in [10]. Briefly, mGlu2^−/−^ mice were crossed with mGlu3^−/−^ mice to generate double heterozygote offspring. Double heterozygotes were crossed to generate double knockout (1:16) and wild-type (WT; 1:16) mice. To avoid excessive animal wastage, separate lines of WT and mGlu2/3^−/−^ mice were established.

### Tissue preparation for homogenate studies

Mice were culled by cervical dislocation. Frontal cortex and striatum were dissected out, snap frozen in isopentane on dry ice and stored at −80°C. For the study of mGlu2/3^−/−^ mice, 16 double knockouts (8 male, 8 female) and 16 WT mice (8 male, 8 female), aged 8–11 months, were used. For the studies of mGlu2^−/−^ and mGlu3^−/−^ mice, 8 males of each type were compared with 8 male littermate controls, aged 11–12 months. Final samples sizes were smaller for some analyses, either due to abnormal chromatograms, or due to outliers.

Immediately prior to HPLC analysis, tissue was removed from the freezer, weighed and homogenised in 0.06M perchloric acid (PCA) with a polytron homogeniser for 20 seconds. Samples were centrifuged for 5 minutes at 14,000 rpm on a desktop centrifuge at 4°C and the supernatant collected and filtered (using a 13 mm syringe filter, Whatman). Samples were handled on ice and run as soon as possible to prevent degradation. Monoamine concentration was measured using HPLC and normalised to the tissue weight of each sample.

### Tissue preparation for tissue punch study

15 mGlu2/3^−/−^ (8 male, 7 female), and 12 WT (7 male, 5 female) mice, aged 6 – 11 months, were culled by cervical dislocation and the whole brain snap frozen in liquid nitrogen and stored at −80°C. Coronal brain sections containing the NAc and striatum were cut on a cryostat (thick sections were produced by pressing the advance button 20 times before cutting each section), collected on untreated glass slides, and stored at −80°C.

Immediately prior to HPLC analysis, slides containing 2 to 3 brain sections for each mouse were placed on dry ice and two tissue punches from each hemisphere were taken, one from dorsolateral striatum and one from ventral striatum containing mostly NAc, resulting in a total of 4 punches per section. Approximately 8 punches per brain region per mouse were taken. A 0.5 mm diameter sample corer (Fine Science Tools 18035–50) was used to make the tissue punch. Punches were ejected into 200 μl of ice cold 0.06M PCA, lysed by placing in an ice cold ultrasonic bath for 1min and centrifuged at 13,000 rpm for 10min in a desk top centrifuge at 4°C. The supernatant was removed, filtered and analysed with HPLC. Protein pellets were collected and stored at −80°C. The total protein content in each sample was determined using Bradford assay; samples were diluted with 5 μl of 1M NaOH, solubilised and 20 μl of ddH_2_O added and then Bradford assay run as per standard protocol (Sigma, UK). Monoamine concentrations were normalised to the protein content as measured above.

### HPLC detection

Immediately after filtration, levels of dopamine, noradrenaline, 5-HT, DOPAC, HVA, MHPG and 5-HIAA were measured using HPLC and electrochemical detection. Analytes were separated by injecting 50 μl of sample into a Microsorb C18 column (100 × 4.6 mm column, 3 μm C18 Microsorb particles, Varian Inc) and a mobile phase containing 120 mM NaH_2_PO_4_, 2 mM NaCl, 0.1 mM EDTA, 2 mM OSA (1-Octanesulphonic acid sodium) and 15% (v/v) methanol, pH 3.71, at a flow rate of 1 ml/min (Jasco pump, Jasco, UK). Analytes were electrochemically detected using an ANTEC Decade II amperometric detector, (set at 28°C [first double knockout experiment]) or 36°C (single knockout and tissue punch experiments), Antec Leyden, Zoeterwoude, The Netherlands), which was equipped with an ISAAC flow cell (Antec Leyden, Zoeterwoude, The Netherlands) operated at +0.6 V with an Ag/AgCl reference electrode. Chromatograms were processed by ChromPass software (Jasco, UK).

### Data analysis

We first checked for outliers, defined as being >3× outside the interquartile range, and assessed data with the D’Agostino and Pearson omnibus K2 normality test. Where data were suitable for parametric analysis, we used one-way ANOVA with genotype and sex as dependent factors for the double knockout mice experiments, and t-tests for the single knockouts (since only males were studied). When data were not normally distributed, the Mann–Whitney U test was used; in the event, the latter was required for analyses of cortical dopamine, DOPAC and 5-HIAA in the mGlu2/3^−/−^ mice, for cortical DA and 5-HT in mGlu2^−/−^ mice, and for striatal DOPAC in the tissue punch study. Analyses were conducted using SPSS v20 and Graphpad Prism 5. All tests were 2-tailed.

## Abbreviations

DOPAC: 3,4-Dihydroxyphenylacetic acid; MHPG: 3-Methoxy-4-hydroxyphenylglycol; 5-HIAA: 5-Hydroxyindoleacetic acid; HPLC: High performance liquid chromatography; HVA: Homovanillic acid; mGlu3−/−: mGlu3 knockout; mGlu2−/−: mGlu2 knockout; mGlu2/3−/−: mGlu2/3 double knockout; NAc: Nucleus accumbens; VTA: Ventral tegmental area; WT: Wild-type.

## Competing interests

JNCK was an employee of GlaxoSmithKline.

## Authors’ contributions

PJH, DMB, JNCK, TAL designed the study. TAL and PJH drafted the manuscript. DMB, TS, EMT critically reviewed the manuscript. TAL carried out the study. TB helped with tissue punch experiments. EMT trained TAL in HPLC. TS supervised experiments. JNCK contributed reagents. All authors read and approved the final manuscript.
